# METTL3-mediated m^6^A modification of circGLIS3 promotes prostate cancer progression and represents a potential target for ARSI therapy

**DOI:** 10.1186/s11658-024-00628-z

**Published:** 2024-08-14

**Authors:** Xiaofeng Cheng, Heng Yang, Yujun Chen, Zhenhao Zeng, Yifu Liu, Xiaochen Zhou, Cheng Zhang, An Xie, Gongxian Wang

**Affiliations:** 1 Department of Urology, The First Affiliated Hospital, Jiangxi Medical College, Nanchang University, Nanchang, 330000 Jiangxi China; 2 Jiangxi Institute of Urology, Nanchang, 330000 Jiangxi China; 3 Department of Urology, Jiangxi Provincial People’s Hospital, The First Affiliated Hospital of Nanchang Medical College, Nanchang, 330000 Jiangxi China

**Keywords:** Prostate cancer, Circular RNA, M^6^A modification, ARSI therapy

## Abstract

**Background:**

Circular RNAs (circRNAs) have been shown to be involved in tumorigenesis and progression. However, the role of circGLIS3 (hsa_circ_0002874) in prostate cancer (PCa) has yet not been reported.

**Methods:**

Candidate circRNA were determined through comprehensive analysis of public datasets, PCa cell lines, and tissues data. A series of cellular functional assays, including CCK-8, colony formation, wound healing, and transwell assays were performed. Subsequently, RNA sequencing, RNA immunoprecipitation, methylated RNA immunoprecipitation, microRNA pulldown, luciferase reporter assay, and western blot were used to explore the underlying molecular mechanisms. Moreover, the xenograft tumor mouse model was established to elucidate the function of circGLIS3.

**Results:**

CircGLIS3, derived from exon 2 of the parental GLIS3 gene, was identified as a novel oncogenic circRNA in PCa that was closely associated with the biochemical recurrence. Its expression levels were upregulated in PCa tissues and cell lines as well as enzalutamide high-resistant cells. The cellular functional assays revealed that circGLIS3 promoted PCa cell proliferation, migration, and invasion. METTL3-mediated *N*^6^-methyladenosine (m^6^A) modification maintained its upregulation by enhancing its stability. Mechanically, CircGLIS3 sponged miR-661 to upregulate MDM2, thus regulating the p53 signaling pathway to promote cell proliferation, migration, and invasion. Furthermore, in vitro and in vivo experiments, the knockdown of circGLIS3 improved the response of PCa cells to ARSI therapies such as enzalutamide.

**Conclusions:**

METTL3-mediated m^6^A modification of circGLIS3 regulates the p53 signaling pathway via the miR-661/MDM2 axis, thereby facilitating PCa progression. Meanwhile, this study unveils a promising potential target for ARSI therapy for PCa.

**Supplementary Information:**

The online version contains supplementary material available at 10.1186/s11658-024-00628-z.

## Background

Prostate cancer (PCa) is the most common malignancy and the second leading cause of cancer-related death among males in Western countries [1]. Since the seminal work of Huggins and Hodges, androgen deprivation therapy (ADT) has been the cornerstone for the management of aggressive PCa [2]. Despite the high initial response rate of ADT, almost all patients inevitably progress into castration-resistant prostate cancer (CRPC) within 3 years [3]. Androgen receptor (AR) signaling inhibitors (ARSI) therapy, such as enzalutamide, were applied to CRPC treatment, yielding significant prognostic improvements [4, 5]. However, the improvement is short-lived, as CRPC ultimately develops drug resistance, due to AR splice variants or bypass activation [6]. Hence, there is an urgent need to delve into the underlying mechanisms to pave the way for innovative therapeutic strategies for PCa.

Circular RNAs (circRNAs) are a novel class of small noncoding RNAs with a covalently single-stranded loop structure, generated by direct back splicing or exon skipping of precursor messenger RNA (mRNA) [7, 8]. Previously, circRNAs were incorrectly considered to be byproducts of splicing errors with little or no biological function [9]. Accumulating studies have demonstrated that circRNAs participate in tumorigenesis and development in a variety of tumors, including PCa [10]. Given its distinctive configuration, circRNAs emerge as promising molecular markers and potential vulnerabilities for therapeutic strategies. Moreover, with the advancement of next-generation sequencing, most circRNAs have been discovered and identified, which provide new perspectives into the etiological mechanisms of human diseases, such as cancer. Research on circRNAs has grown exponentially over the years, which provided valuable insights into tumorigenesis, but it is still lacking particularly in PCa.

*N*^6^-methyladenosine (m^6^A) modification is the most abundant chemical modification of mRNA known in eukaryotic cells [11]. It is a dynamic and reversible process that regulates RNA transcription, processing, splicing, stability, and translation [12]. Several studies have shown that m^6^A modification contributes to PCa progression. The increased overall m^6^A level in CRPC relative to castration-sensitive PCa serves as evidence [13]. METTL3 knockdown remarkably alters the basal and androgen-regulated transcriptome in PCa [14]. In addition, the m^6^A modification occurs not only on mRNAs but also on noncoding RNA types, including circRNA [15, 16]. Numerous studies focused on the m^6^A modification of mRNA [17]. However, circRNAs display the m^6^A modification pattern that is distinct from that of mRNAs [18]. For some noncoding RNAs, especially for circRNAs, the effects of m^6^A on its biology remain to be further elucidated.

In this study, through comprehensive retrieval and analysis of the public datasets (including GSE155792 and GSE113120) of the Gene Expression Omnibus (GEO) database, we selected circGLIS3 (genomic location: chr9:4286037–4286523; circBase ID: has_circ_0002874) as the candidate gene, which was strongly related to the biochemical recurrence (BCR)-free survivals in PCa. Mechanistically, circGLIS3, which was localized mainly in the cytoplasm, modulates the p53 signaling pathway, promoting PCa progression through the miR-661/MDM2 axis. Meanwhile, its stability was regulated by the METTL3-mediated m^6^A modification levels. Additionally, circGLIS3 represents a promising potential target for ARSI therapy.

## Materials and methods

### Patient selection and samples collection

PCa and adjacent normal tissue specimens were obtained from 15 cases at the First Affiliated Hospital of Nanchang University between June 2017 and June 2020. The study meticulously adhered to inclusion criteria, including (1) patients who underwent radical prostatectomy and (2) independent confirmation of the pathological diagnosis as prostate adenocarcinoma by two pathologists. Exclusion criteria encompassed: (1) patients who had received any form of antitumor therapy before surgery, including neoadjuvant endocrine therapy, radiotherapy, or chemotherapy and (2) the presence of distant metastases. All tissue specimens were promptly preserved in liquid nitrogen or −80 °C after surgery. Informed consent was signed by each patient before surgery. This study was approved by the Ethics Committee of the First Affiliated Hospital of Nanchang University (approval no. 075[2017]). All animal experiments were approved by the Animal Care Committee of the First Affiliated Hospital of Nanchang University (approval no. CDYFY-IACUC-202302QR031).

### Cell culture

The normal primary prostate epithelial cell lines, HPrEC, and human PCa cell lines, including VCAP and C4-2, were procured from the American Type Culture Collection (Manassas, USA). Additionally, human PCa cell lines, involving DU145, PC3, and 22RV1, were obtained from Procell Life Science and Technology Co. Ltd. (Wuhan, China). DU145, 22RV1, and C4-2 cells were cultured in RPMI-1640 medium (Gibco, USA), PC3 cells in F12K medium (BOSTER, China), VCaP cells in Dulbecco’s modified Eagle medium (DMEM) (Gibco, USA), and HPrEC cell in prostate epithelial cell basal medium supplemented with growth factors (ATCC PCS-440-040). All the media were supplemented with 10% fetal bovine serum (FBS) (BI Biological Industries) and 1% penicillin–streptomycin solution (New Cell and Molecular Biotech Co., Ltd, China). All cell lines were incubated at 37 °C with 5% CO_2_ under saturated humidity.

### Oligo RNA, plasmids, and lentivirus

Small interfering RNA (SiRNA) of circGLIS3, MDM2, and METTL3 was designed by RiboBio (Guangzhou, China). MicroRNA (miRNA)-661 mimics and inhibitors and plasmids overexpressing circGLIS3 were synthesized by GenePharma (Suzhou, China). The above oligo RNAs were transiently transfected using Lipofectamine 3000 (Invitrogen) according to the manufacturer’s instructions. Lentivirus overexpressing circGLIS3 and lentivirus carrying encoding short hairpin RNAs (shRNA) were constructed by GenePharma (Suzhou, China). The stable cell line was established by treating lentivirus-transfected cells with 6 μg/ml puromycin for 24 h. miR-661 biotin-labeled probes were synthesized by GenePharma (Suzhou, China). Bulge-loop™ miRNA quantitative real-time polymerase chain reaction (qRT–PCR) Primer Set (one RT primer and a pair of qPCR primers for each set) specific for U6, miR-1200, miR-661, and miR-663b were designed by RiboBio(Guangzhou, China). Other gene primers were synthesized by Sangon Biotech (Shanghai, China). Sequence information is available in Supplementary Table S1.

### Quantitative real-time polymerase chain reaction (qRT‒PCR)

Total RNA was isolated using TRIzol reagent (DP424, TIANGEN Biotech, Beijing, China) and reversed transcribed into complementary DNA (cDNA) with the FastKing RT Kit (with gDNase) (KR116, TIANGEN Biotech, Beijing, China) according to the manufacturer’s description. qRT–PCR was performed in triplicate with perfectStart Green qPCR SuperMix (AQ601, TransGen Biotech, Beijing, China). The 2^−ΔΔCT^ method was used to calculate the relative expression level, which was normalized to the internal reference gene β-actin or U6.

### RNA stability assays

To assess the stability of circRNAs, 4 μg of total RNA was subjected to digestion with or without 1 U/μg RNase R (R0301, Geneseed, Guangzhou, China) at 37 °C for 15 min. After inactivating the RNase R enzyme at 70 °C for 10 min, the product was directly utilized for reverse transcription. In addition, 5 μg/ml or 400 μg/ml actinomycin D (HY-17559, MedChemExpress, USA) was added to the medium to culture PCa cells. At the specific time points, the cultured cells were harvested to isolate total RNA, which was ultimately used to perform qRT–PCR with equal volumes.

### Nucleocytoplasmic RNA isolation

To determine the subcellular localization of circGLIS3, nuclear and cytoplasmic RNA isolation was conducted using the NE-PER™ nuclear and cytoplasmic extraction reagents kit (78833, Thermo Fisher Scientific, Rockford, USA), following the manufacturer’s descriptions. U6 and β-actin were utilized as gene references in the nucleus and cytoplasm, respectively, to evaluate the relative distribution of RNA.

### RNA in situ hybridization (ISH) and fluorescence in situ hybridization (FISH) assay

ISH assays were carried out to detect the circGLIS3 expression in PCa tissue microarray (Shanghai Outdo Biotech, Shanghai, China) using a digoxigenin-labeled probe. The probe and ISH kit were designed and synthesized from BOSTER (Wuhan, China). In addition, the FISH assay was performed using the Ribo™ Fluorescent In Situ Hybridization Kit (C10910, RiboBio). Cy3-labeled circGLIS3 probe was purchased from GenePharma (Suzhou, China). For the FISH assay, DU145 and PC3 cells were planted in 24-well plates containing round coverslip before 24 h, fixed with 4% paraformaldehyde, and perforated with precooled 1% Triton. Cells were then incubated with prehybridization buffer for 30 min at 37 °C and then overnight at 37 °C with hybridization buffer mixed with a 20 μM circGLIS3 probe. Cells were then washed using a wash buffer containing 4× Saline Sodium Citrate (SSC), 0.1% Tween-20, 2× SSC, and 1× SSC. Eventually, the nuclei were stained with DAPI. The subcellular localization of circGLIS3 was visualized using the Leica confocal imaging system.

### Western blot assay and antibodies

Total proteins were extracted using precooled RIPA lysis buffer (APExBIO, USA) containing protease and phosphatase inhibitors. Isolated proteins were subsequently transferred onto polyvinylidene fluoride membranes, which were then immersed in 5% nonfat milk for 2 h. The membranes were incubated overnight at 4 °C with the primary antibody and then with the secondary antibody for 1.5 h at room temperature. Anti-GAPDH (GB11002) and anti-Ki67 (GB121141) antibodies were purchased from Servicebio. anti-MDM2 (no. 86934) antibody was purchased from Cell Signaling Technology. Anti-p53 (60283-2-Ig), anti-AGO2 (67934-1-Ig), anti-METTL3 (15073-1-AP), and anti-IgG(B900620; 30000-0-AP) antibody were obtained from proteintech (Wuhan, China). Anti-AR (sc-7305) antibody was available from Santa Cruz. The anti-m^6^A (202003) antibody was acquired from Synaptic Systems. Secondary antibodies include anti-mouse and anti-rabbit antibodies from ZSGB-BIO.

### Cell proliferation, colony formation, invasion, and migration assays

Cell Counting Kit-8 (CCK-8) assay was conducted to assess cell proliferation. A total of 5–8 × 10^3^ cells per well were seeded in 96-well plates. After 6, 24, 48, 72, and 96 h of cell culture, 10 μL of CCK-8 solution was added to each well and after 2 h of incubation, and the absorbance value at 450 nm was then measured for each well. For the clone forming assays, 1 × 10^3^ cells per well were seeded in six-well plates. After 2 weeks of cell culture, 4% paraformaldehyde (Servicebio, Wuhan, China) and 1% crystal violet (Solarbio, Beijing, China) solution were used to fix and stain cells, respectively. For invasion assays, the upper chamber was covered with Matrigel (Corning, NY). A total of 5–6 × 10^3^ cells per well were seeded and cultured in the upper chamber of the transwell under FBS-free conditions for 24 or 48 h, while the lower chamber was maintained in a high-FBS condition (20% FBS). The invaded cells on the compartment were fixed and stained using the same methods as mentioned above. For wound healing assays, cells cultured to confluence in six-well plates were scratched using a 200 μl aseptic tip and then cultured under FBS-free conditions. After allowing the cell to migrate or close the gap, images were captured at 0 and 36 h, respectively.

### Apoptosis and cell cycle assays

For apoptosis assays, the V-FITC/PI cell apoptosis detection kit (no. FA101-01, TransGen, Beijing, China) was used to detect apoptotic cells. Similarly, for cell cycle assays, the cell cycle and apoptosis analysis kit (C1052, Beyotime, Shanghai, China) was applied to assess cell cycle phase distribution. All the above procedures were conducted following the manufacturer’s instructions.

### RNA immunoprecipitation (RIP) and methylated RNA Immunoprecipitation (meRIP) assays

RIP assays were performed using a PureBinding^®^ RNA Immunoprecipitation Kit (Geneseed Biotech, P0101). For RIP assays, the cell lysates were immunoprecipitated with A/G magnetic beads conjugated with anti-AGO2 or IgG overnight at 4 °C. The enriched RNA was then eluted from the magnetic beads and used to be detected by qPCR. In the MeRIP assay, total RNA was extracted from DU145 and PC3 cells, immunoprecipitated with anti-m^6^A antibody for 2 h at 4 °C, incubated with magnetic beads at 4 °C overnight, and then eluted with elution buffer for 60 min at 50 °C to obtain the m^6^A-modified RNA. The enriched RNAs were isolated with a phenol–chloroform–isoamyl–alcohol (25:24:1) mixture and then quantified by qRT–PCR.

### Luciferase reporter assay

The circGLIS3 and MDM2 wild-type and mutant-type sequences, designed to target the predicted binding sites, were cloned into a dual-luciferase reporter vector of pSI-Check2. Wild-type and mutant-type plasmids were purchased from Hanbio Biotechnology Co., Ltd. The plasmid was cotransfected with miR-661 mimics or negative control (NC) into HEK-293T cells at the logarithmic growth stage. After 48 h of transfection, the Dual-Luciferase Reporter Assay Kit (Hanbio, Shanghai, China) was adopted to detect the activity of firefly luciferase and Renilla luciferase according to the manufacturer’s instructions.

### miRNA pulldown assay

For the miRNA pull-down assay, the biotin-labeled RNA probes were utilized to capture the target RNA binding to the miRNA. The biotin-miR-661 or biotin-NC pull-down probes were designed by Genepharma (Shanghai, China) and incubated with Dynabeads™ MyOne™ Streptavidin C1 beads (65002, Thermo Fisher Scientific, Rockford, USA). DU145 and PC3 cell lysate were incubated with streptavidin magnetic beads in the vertical rotator at 4 °C for 3 h. The RNA–bead complex then was washed with lysis buffer and further eluted with elution buffer to obtain the enriched RNA. qRT–PCR was applied to evaluate the relative enrichments of circGLIS3 and MDM2.

### Immunohistochemistry (IHC)

For the IHC assay, the primary antibodies, including anti-Ki67, anti-MDM2, and anti-p53, were applied to incubate the paraffin sections at 4 °C overnight. Then, slices were incubated with a secondary antibody at room temperature for 30 min and dyed with diaminobenzidine (DAB) tetrahydrochloride, In addition, nuclei were detected by hematoxylin staining. The pathological section scanner was used to scan the slices.

### Xenograft experiment

Four-week-old male nude BALB/c mice were randomly divided into two groups and subcutaneously injected with 200 μl PBS containing 5 × 10^6^ DU145 or PC3 cells transfected with shNC or sh-circGLIS3 lentivirus, respectively. The subcutaneous graft tumor mouse model was constructed to investigate the effect of circGLIS3 on tumor growth in vivo. To elucidate the resistance to enzalutamide in vivo, mice inoculated with transplanted tumors were randomized into two groups and again orally administered 10 mg/kg enzalutamide (A3003, APExBIO, USA) or dimethyl sulfoxide (DMSO) for 28 days, respectively. To assess PCa cell responses to enzalutamide, the half-maximal inhibitory concentration (IC50) was measured. During this time, the tumor volume was detected every 5 days and calculated (volume = 0.5 × length × width^2^). Additionally, the tumor weight was measured when the mice were sacrificed.

### Sequencing data and public data analysis

Total RNA from three pairs of siRNA-circGLIS3 and siRNA-NC PC3 cells was extracted, and after quality control, it was sequenced on the Illumina platform at Oebiotech company (Shanghai, China). The differentially expressed genes were obtained with the criteria of *q*-value < 0.001 and |log_2_ fold change (FC)|> 0.2 via using the OECloud tools at https://cloud.oebiotech.com. Functional enrichment analyses were conducted using the “clusterProfiler” package to elaborate on the potential pathways. Public expression data involving GSE113120, GSE155792, GSE113124, GSE65061, GSE94767, GSE118959, DKFZ-PRAD, and TCGA-PRAD were available in the GEO, the cBioPortal, The Cancer Genome Atlas (TCGA), and the Genotype-Tissue Expression (GTEx) database. Clinical information and circRNA annotation for GSE113120 were acquired from Sujun Chen et al. [19] and the circBank database, respectively. All data processing and visualization were conducted using R software (version 4.3.1). The circGLIS3, miR-661, and METTL3 expression levels were stratified into high and low expression according to the optimal threshold determined by the “*survminer*” package. The “*survival*” R package was installed to assess the BCR-free survival through the Kaplan–Meier survival curve with the log-rank test. Furthermore, the Pearson correlation coefficient was calculated using the “*stats*” package.

### Statistical analysis

All continuous variables were presented as mean ± standard deviation (SD), while categorical variables as numbers and proportions. All statistical analysis and visualization were performed by GraphPad Prism (version 10.0), besides data from public databases and sequencing. Student’s *t*-test, Mann–Whitney *U* test, and the chi-squared test were appropriately selected to detect the significance. A *p*-value of less than 0.05 was used as the criterion for statistical significance, with the following notations: * for *p* < 0.05, ** for *p* < 0.01, *** for *p* < 0.001, and **** for *p* < 0.0001.

## Results

### CircGLIS3 is upregulated in PCa and its characteristics

**Fig. 1:**
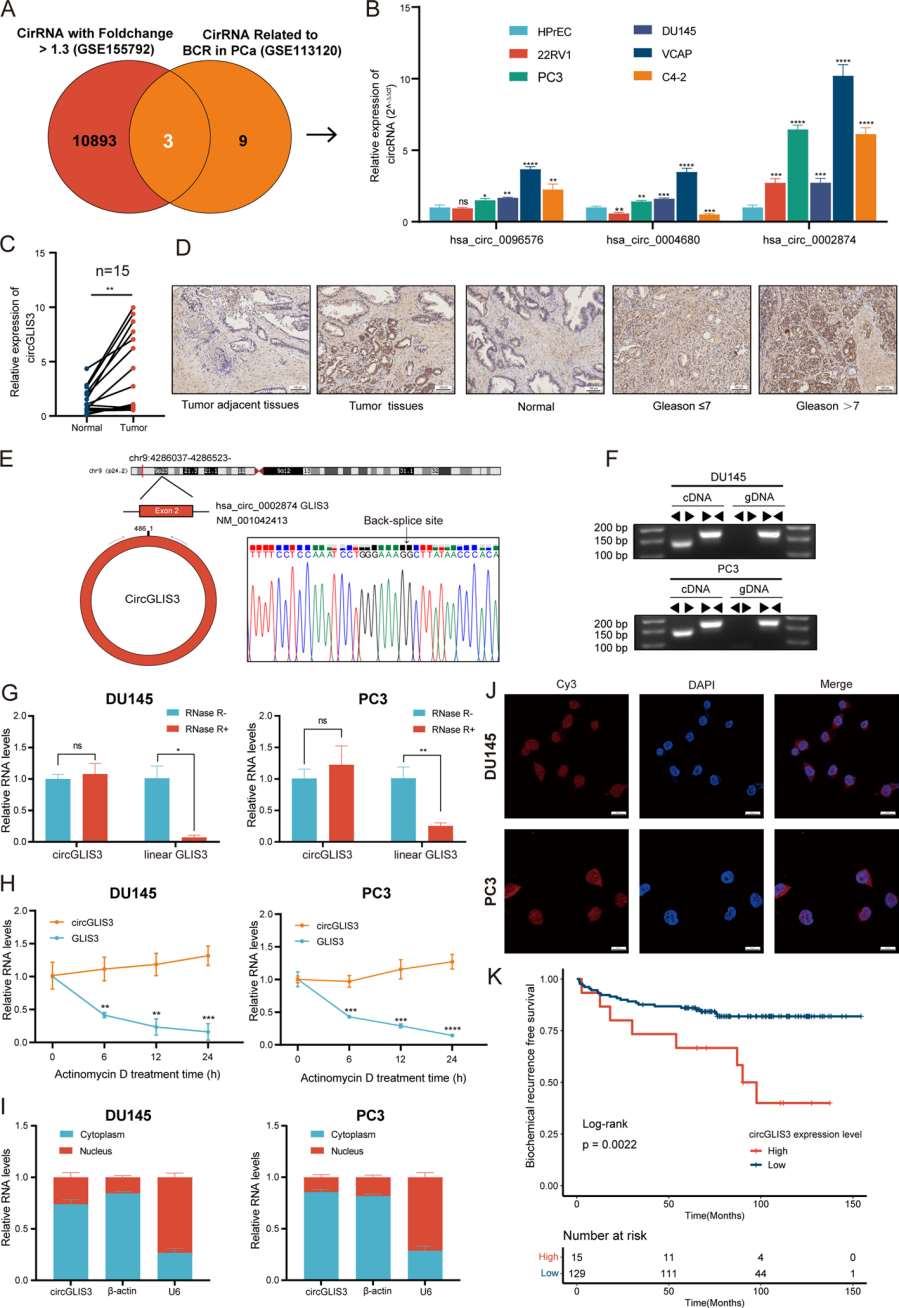
Selection of candidate circRNAs and characteristics of circGLIS3 in PCa. **A** Venn diagram of circRNAs related to BCR in GSE113120 and with FC > 1.3 in GSE155792. **B** The relative expression of candidate circRNAs in PCa or normal cell. **C** The relative expression of circGLIS3 in PCa tissues and adjacent normal tissues. **D** The expression level of circGLIS3 was detected by in situ hybridization on PCa tissue microarray, showing that circGLIS3 was upregulated in PCa tissues compared with adjacent tissues, and such upregulation was positively correlated with a higher Gleason score. **E** The closed-loop structure of circGLIS3 was validated by Sanger sequencing. **F** Divergent and convergent primers amplification assays. **G**, **H** The RNase R and actinomycin D assay confirmed that circGLIS3 was more stable than linear GLIS3. **I**, **J** RNA fractionation and FISH assays revealed that circGLIS3 RNA was mainly located in the cytoplasm. **K** The Kaplan–Meier survival curve revealed the association between circGLIS3 and the prognosis in PCa. The data were presented as mean ± SD. *p < 0.05, **p < 0.01, ***p < 0.001, and ****p < 0.0001

To determine the vital circRNAs for PCa prognosis, which fulfill the following criteria, FC > 1.3 in GSE155792 and related to BCR in GSE113120 (fragments per kilobase million > 0.5, *p*-value < 0.005 and hazard ratio > 1 in Cox regressions) (Supplementary Table S2). As illustrated in Fig. 1A, hsa_circ_0096576, hsa_circ_0004680, and hsa_circ_0002874 (known as circGLIS3) were identified. CircGLIS3 expression was significantly upregulated in PCa cell lines and tissues (Fig. 1B, C). Moreover, the expression levels of circGLIS3 was further detected in tissue microarray consisting of 64 PCa and nontumor tissues. The result of ISH assay also indicated that circGLIS3 was significantly higher expressed in tumor tissues and those with the advanced clinicopathologic stages such as Gleason score (Fig. 1D). As shown in Fig. 1E, the parental gene GLIS3 was located on chromosome 9, and its exons 2 were generated to circGLIS3 by back-splicing. The closed-loop configuration was ascertained by the Sanger sequencing (Fig. 1E). To further confirm this structure, divergent and convergent primers were designed to amplify the transcripts of GLIS3 from cDNA and genomic DNA (gDNA). Its results indicated that circGLIS3 was only amplified in cDNA but not gDNA using divergent primers (Fig. 1F). RNase R and actinomycin D assays were conducted to assess its stability. As depicted in Fig. 1G, circGLIS3 was more resistant to RNase R than linear GLIS3. Likewise, circGLIS3 was more stable than linear GLIS3 after actinomycin D treatment (Fig. 1H). Concerning its subcellular localization, the nucleocytoplasmic RNA isolation assays revealed that circGLIS3 was located mainly in the cytoplasm, which was also validated by the FISH assay (Fig. 1I, J).

In the localized intermediate-risk PCa (Canadian Prostate Cancer Genome Network, CPC-GENE) cohort, circGLIS3 expression was closely associated with the BCR (Supplementary Table S2). As illustrated in Fig. 1K, the Kaplan–Meier survival curve showed that patients with high circGLIS3 expression exhibited worse BCR-free survival than those with low expression. In multivariate Cox regression, both the Gleason score and circGLIS3 expression were identified as independent prognostic indicators. (Table 1). In brief, the above results indicated that circGLIS3 serves as a promising biomarker in PCa.

**Table 1:** Univariate and multivariate Cox regression analysis for BCR in CPC-GENE cohorts

Variables	Univariate analysis		Multivariate analysis	
	HR (95% CI)	*p*-value	HR (95% CI)	*p*-value
Age				
< 62	Reference			
≥ 62	1.158 (0.558–2.400)	0.694		
PSA				
< 4	Reference			
≥ 4	1.165 (0.353–3.851)	0.802		
T stage				
T1	Reference		Reference	
T2	1.769 (0.851–3.677)	0.127	1.631 (0.766–3.473)	0.204
Gleason score				
≤ 7	Reference		Reference	
> 8	6.555 (1.530–28.080)	0.011	6.444 (1.413–29.385)	0.016
CircGLIS3 expression level				
Low	Reference		Reference	
High	3.403 (1.506–7.690)	0.003	3.772 (1.648–8.630)	0.002

*HR* hazard ratio, *95% CI* 95% confidence interval, *PSA* Prostate-specific antigen

### CircGLIS3 promotes PCa proliferation, migration, and invasion in vitro

**Fig. 2:**
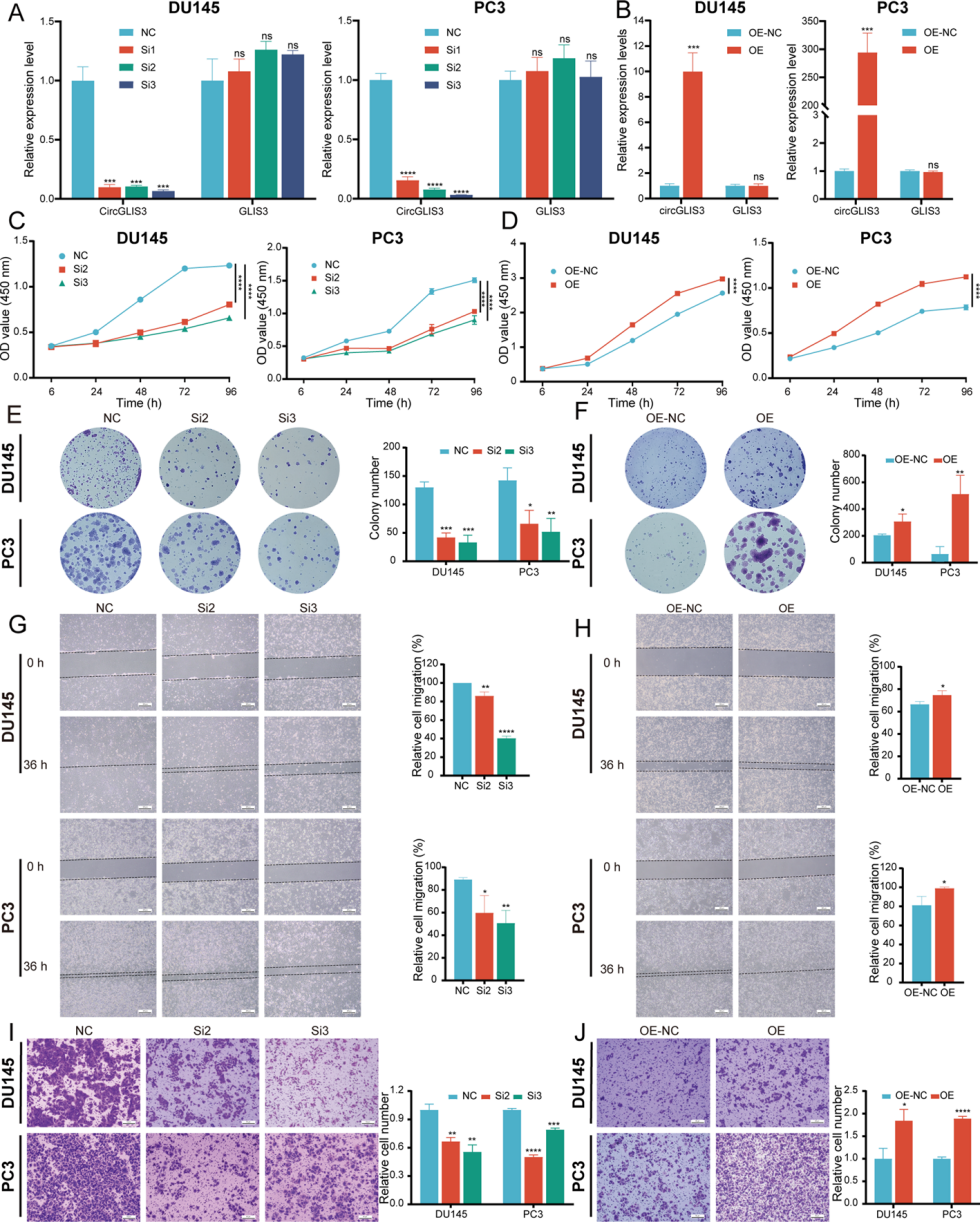
CircGLIS3 contributed to PCa cell proliferation, migration, and invasion. **A** qRT–PCR analysis of the relative circGLIS3 and linear GLIS3 expression after circGLIS3 knockdown in DU145 and PC3 cells. **B** The relative circGLIS3 and linear GLIS3 expression were detected with qRT–PCR after circGLIS3 overexpression. **C**–**F** In CCK-8 and colony formation assays, suggesting that circGLIS3 promoted the growth of PCa cells. **G**–**J** The wound healing (**G**, **H**) and transwell (**I**, **J**) assays revealed the role of circGLIS3 in contributing to migration and invasion. The data were presented as mean ± SD. *p < 0.05, **p < 0.01, ***p < 0.001, and ****p < 0.0001

To investigate the cellular functions of circGLIS3, knockdown siRNAs and overexpressed plasmids or lentivirus were transfected into DU145 and PC3 cells, respectively. circGLIS3 expression was significantly downregulated or upregulated, respectively, while its parental gene expression was unchanged (Fig. 2A, B). The CCK-8 assay demonstrated that circGLIS3 knockdown inhibited cell proliferation, while its upregulation accelerated cell growth (Fig. 2C, E). Similarly, the results of the plate colony formation assay also supported this finding (Fig. 2D, F). Meanwhile, the flow cytometry assay indicated that silencing circGLIS3 markedly inhibited the S phase of the cell cycle and stimulated apoptosis of DU145 and PC3 cells (Supplementary Fig. 1A–D). In the wound healing assay, the relative healing areas were changed after silencing or overexpressing circGLIS3, suggesting that circGLIS3 promoted PCa cell migration (Fig. 2G, H). In the transwell assay, downregulating circGLIS3 hindered cell invasiveness (Fig. 2I). On the contrary, overexpressing circGLIS3 contributed to the invasiveness of DU145 and PC3 cells (Fig. 2J). Taken together, the abovementioned results suggested that circGLIS3 contributed to PCa cell proliferation, migration, and invasion in vitro.

### METTL3-mediated m^6^A modification stabilizes the expression of circGLIS3

**Fig. 3:**
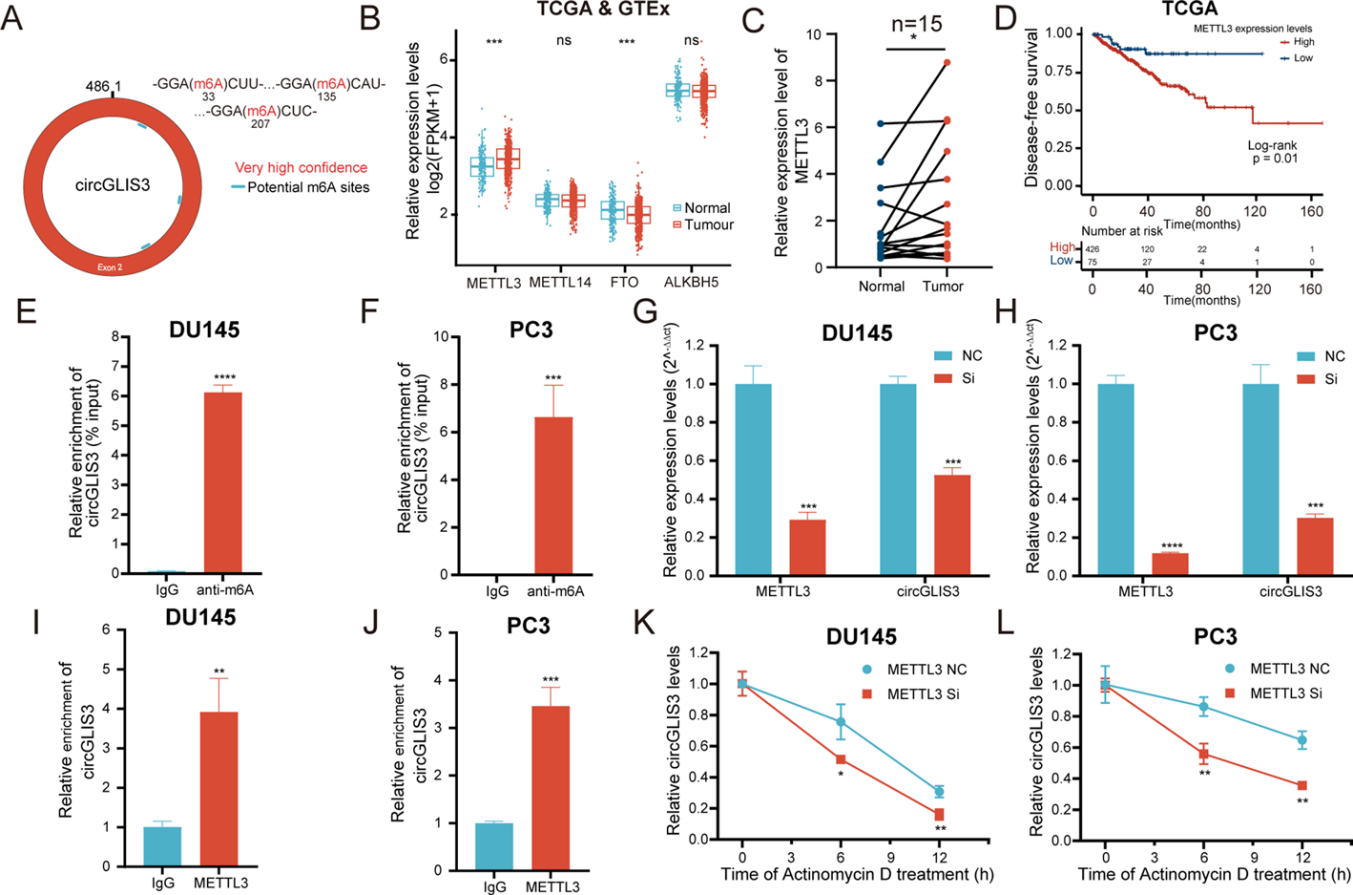
m^6^A modification of circGLIS3. **A** m^6^A modification site of circGLIS3 was predicted in the SRAMP database with very high confidence. **B** Expression levels of m^6^A modification-related genes in TCGA and GETx databases. **C** The relative expression levels of METLL3 in PCa tissues and adjacent normal tissues. **D** The Kaplan–Meier curve analysis revealed the prognosis value of METTL3 in the TCGA-PRAD cohort. **E–F** MeRIP assays demonstrated that circGLIS3 harbors m^6^A modifications in DU145 and PC3 cells, respectively. **G**, **H** The relative expression of circGLIS3 was significantly reduced after silencing METTL3 in DU145 and PC3 cells, respectively. **I**, **J** RIP assays showed that the METTL3 protein was directly bound to circGLIS3 in PCa cells, thereby mediating its m^6^A modification. **K**, **L** RNA stability assays indicated that the half-life of circGLIS3 was remarkably shortened after the knockdown of METTL3. The data were presented as mean ± SD. *p < 0.05, **p < 0.01, ***p < 0.001, and ****p < 0.0001

Accumulating evidence has shown that m^6^A modifications participate in epigenetic regulation and RNA stability. To elucidate the upregulation of circGLIS3 in PCa, its potential m^6^A modification sites were predicted using the SRAMP database (https://www.cuilab.cn/sramp). Its result showed three potential m^6^A modification sites with very high confidence in the SRAMP website (Fig. 3A).

To identify potential m^6^A modification genes that interacted with circGLIS3, bioinformatics analysis was conducted using TCGA and GTEx databases. Ultimately, METTL3 and FTO were identified as potential key genes (Fig. 3B). Furthermore, METTL3, a core component of the m^6^A methylase complex, has been demonstrated to promote invasion and metastasis of PCa cells [14, 20]. Its expression was further validated in our PCa cohorts (Fig. 3C) and closely related to the prognosis (Fig. 3D). Hence, we speculated that METTL3 might induce m^6^A modification of circGLIS3. In the meRIP assay, the results ascertained the m^6^A modification in circGLIS3 (Fig. 3E and F). Besides, the silencing of METTL3 notably decreased circGLIS3 expression and its m^6^A levels (Fig. 3G, H and Supplementary Fig. 2A, B). Subsequently, the RIP assay demonstrated that circGLIS3 was significantly enriched in the METTL3 antibody compared with IgG (Fig. 3I and J). Based on this fact, we hypothesized that METTL3-mediated m^6^A modification might affect its stability. RNA stability assays revealed that the half-life of PCa cells transfected with METTL3-siRNA was shorter compared with scramble (Fig. 3K, L). These results suggest that METTL3-mediated m^6^A modification promotes the upregulation of circGLIS3 by maintaining its RNA stability.

### CircGLIS3 functions as a sponge of miR-661

**Fig. 4:**
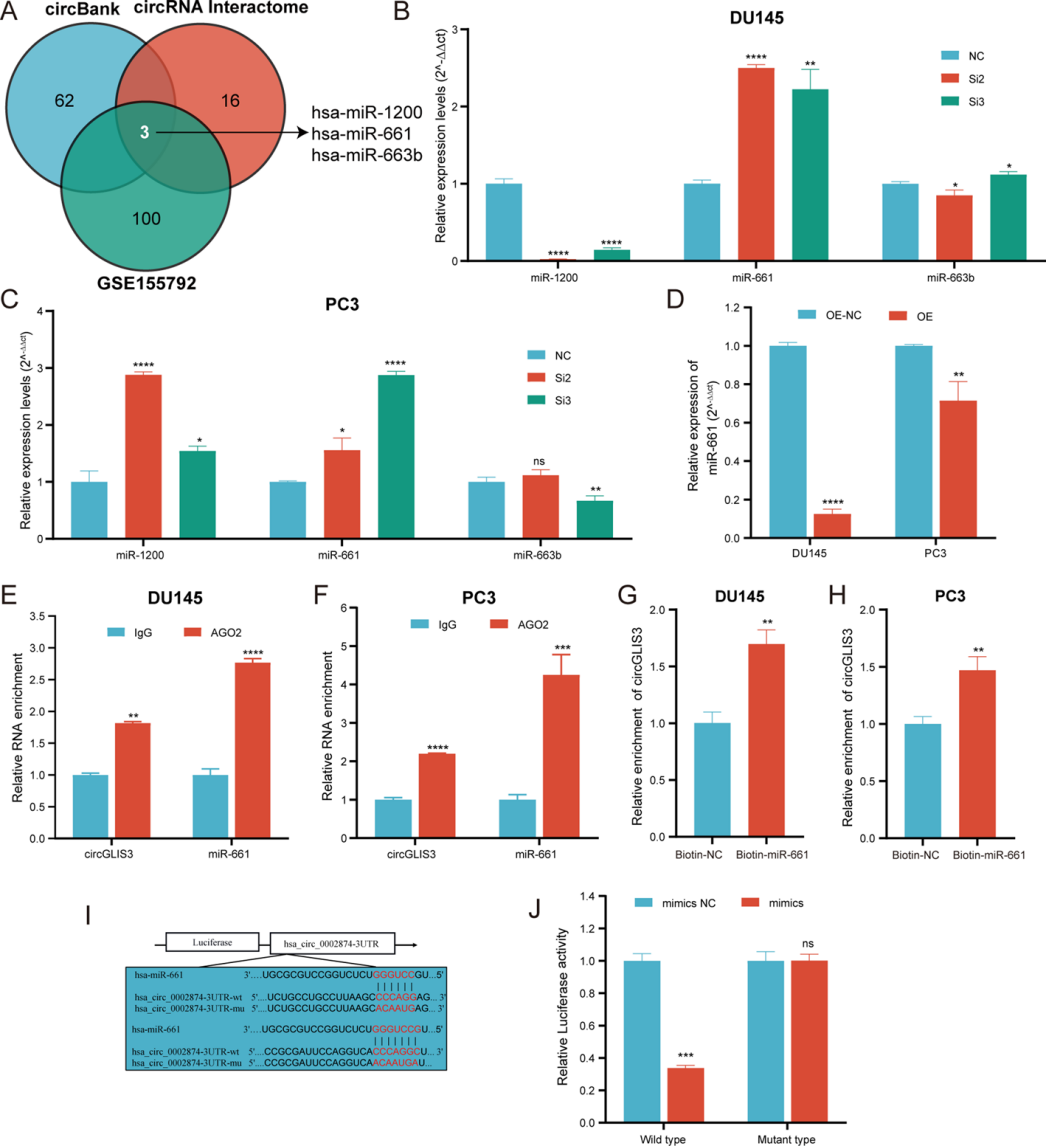
CircGLIS3 served as a sponge for miR-661. **A** The potential candidate miRNA binding on circGLIS3 was predicted according to GSE155792, circbank, and circRNA interactome database. **B**, **C** Relative miRNA expression after downregulation of circGLIS3 in DU145 and PC3 cells, respectively. **D** miR-661 expression was reduced after the overexpression of circGLIS3. **E**, **F** RIP assays revealed that circGLIS3 and miR-661 RNA were directly bound to AGO2 protein in DU145 and PC3 cells. **G**, **H** In miRNA pulldown assays, the enrichment of circGLIS3 was detected in DU145 and PC3 cells. **I** The circGLIS3 luciferase reporter vectors, involving wild-type and mutated-type, were constructed according to the predicted binding sites. **J** The luciferase activities were assessed upon cotransfection with circGLIS3 wild-type or mutated-type plasmid and miR-661 mimics or scramble. The data were presented as mean ± SD. *p < 0.05, **p < 0.01, ***p < 0.001, and ****p < 0.0001

Given that circGLIS3, as an exonic circular RNA, was predominantly located in the cytoplasm, we then hypothesized whether it could act as a miRNA sponge to regulate the target gene expression, thereby promoting PCa progression. Using the online bioinformatics database (circBlank, circRNA Interactome, and GSE155792), miR-1200, miR-661, and miR-6633b were identified, as shown in Fig. 4A. Only miR-661 expression was simultaneously upregulated in both DU145 and PC3 transferred with circGLSI3 siRNAs (Fig. 4B, C). As expected, miR-661 expression was suppressed after circGLIS3 overexpression (Fig. 4D). The RIP assay confirmed the miRNA binding accessibility of circGLIS3, showing that circGLIS3 and miR-661 were notably enriched by anti-AGO2 compared with anti-IgG (Fig. 4E, F). To ascertain their interaction and conjunction, biotin-labeled miR-661 probes were designed to pull down RNA or protein molecules bound to it. The results demonstrated that circGLIS3 interacted with and miR-661 in DU145 and PC3 cells (Fig. 4G, H). Moreover, as illustrated in Fig. 4I, the wild-type and mutated-type circGLIS3 luciferase reporter plasmids were constructed. In the luciferase reporter assay, the luciferase activity was obviously inhibited in cells cotransfected with miR-661 mimics and circGLIS3 wild-type plasmid, whereas it was not altered in cells transfected with a mutant-type plasmid (Fig. 4J). Taken together, circGLIS3 acts as a miRNA sponge to directly bind miR-661 in PCa.

### miR-661 inhibits PCa cell proliferation, migration, and invasion in vitro

**Fig. 5:**
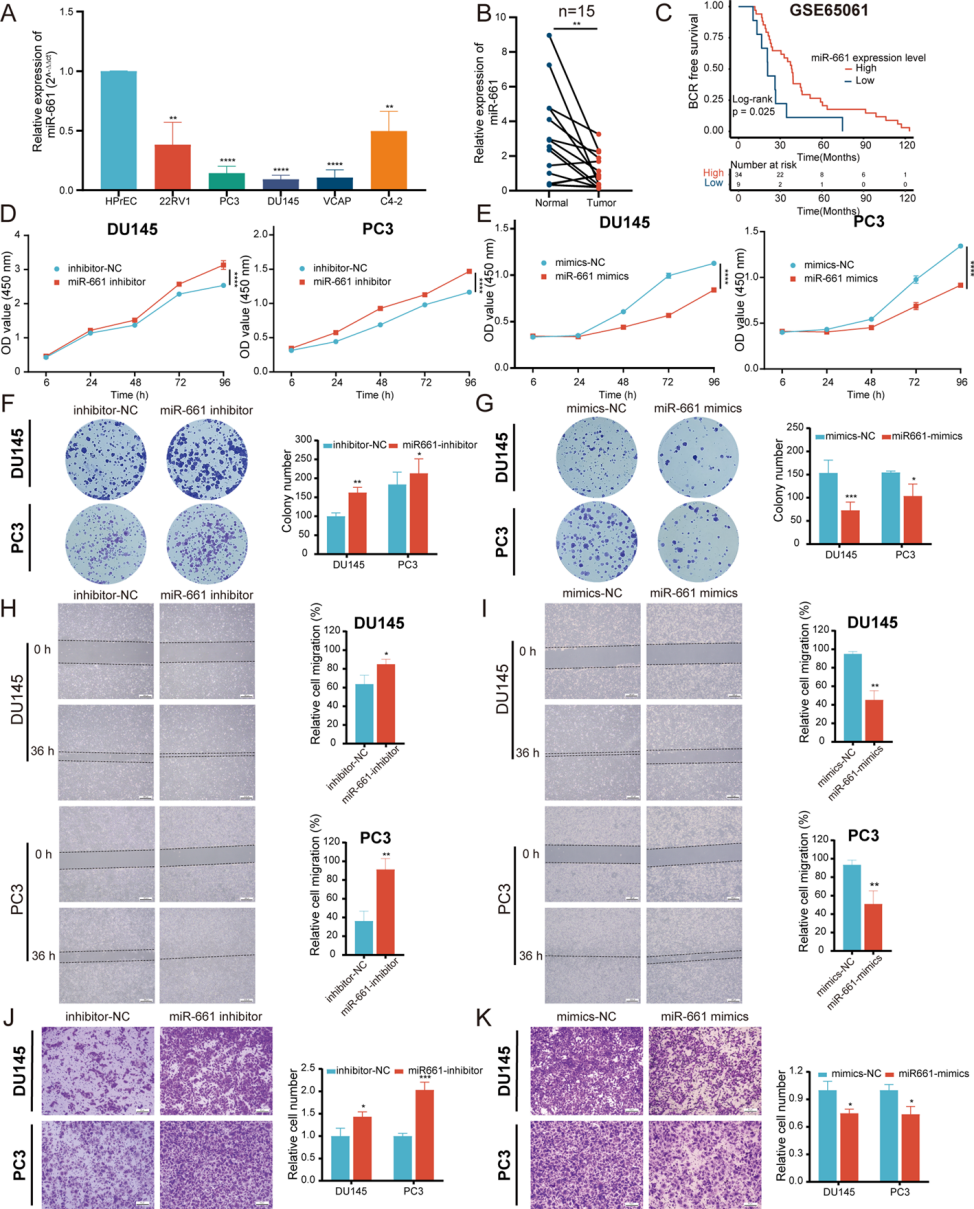
miR-661 inhibited PCa cell proliferation, migration, and invasion. **A**, **B** The relative expression of miR-661 in PCa cell lines (**A**) and tissues (**B**). **C** The Kaplan–Meier survival curve displayed the prognosis value of miR-661 in the GSE65061 cohort. **D**–**G** miR-661 silence promoted PCa cell proliferation, while the miR-661 overexpression suppressed cell viability in CCK-8 and colony formation assays. **H**–**K** In wound healing (**H**–**K**) and transwell (**J**, **K**) assays, miR-661 impaired cell migration and invasiveness. The data were presented as mean ± SD. *p < 0.05, **p < 0.01, ***p < 0.001, and ****p < 0.0001

Currently, the biological function of miR-661 has been elucidated in various tumors, but its role in PCa remains unclear. Its expression then was validated in PCa cell lines and tissues, indicating that it was apparently downregulated (Fig. 5A, B). Low miR-661 expression was associated with poorer BCR-free survival in GSE65061 (Fig. 5C). Besides, its expression was negatively correlated with PSA levels and the T stage, as illustrated in Supplementary Fig. 2C, D. miR-661 mimics and inhibitors were synthesized for overexpression and knockdown of miR-661 expression, respectively, with the inhibitors not significantly altering its expression on PCR because it did not affect miRNA production (Supplementary Fig. 2E–H). In the CCK-8 and plate colony formation assays, overexpressing miR-661 hindered cell growth whereas silencing of miR-661 enhanced the proliferation of DU145 and PC3 cells (Fig. 5D–G). Concurrently, the wound healing assay exhibited notable alterations in the relative migration areas, suggesting miR-661 inhibited PCa cell migration (Fig. 5H and I). The transwell assay also showed that relative cell invasion was reduced with miR-661 overexpression and increased with miR-661 knockdown (Fig. 5J and K). Overall, miR-661 inhibits PCa cell proliferation, migration, and invasion in vitro*.*

### miR-661 reverses circGLIS3-induced PCa progression

**Fig. 6:**
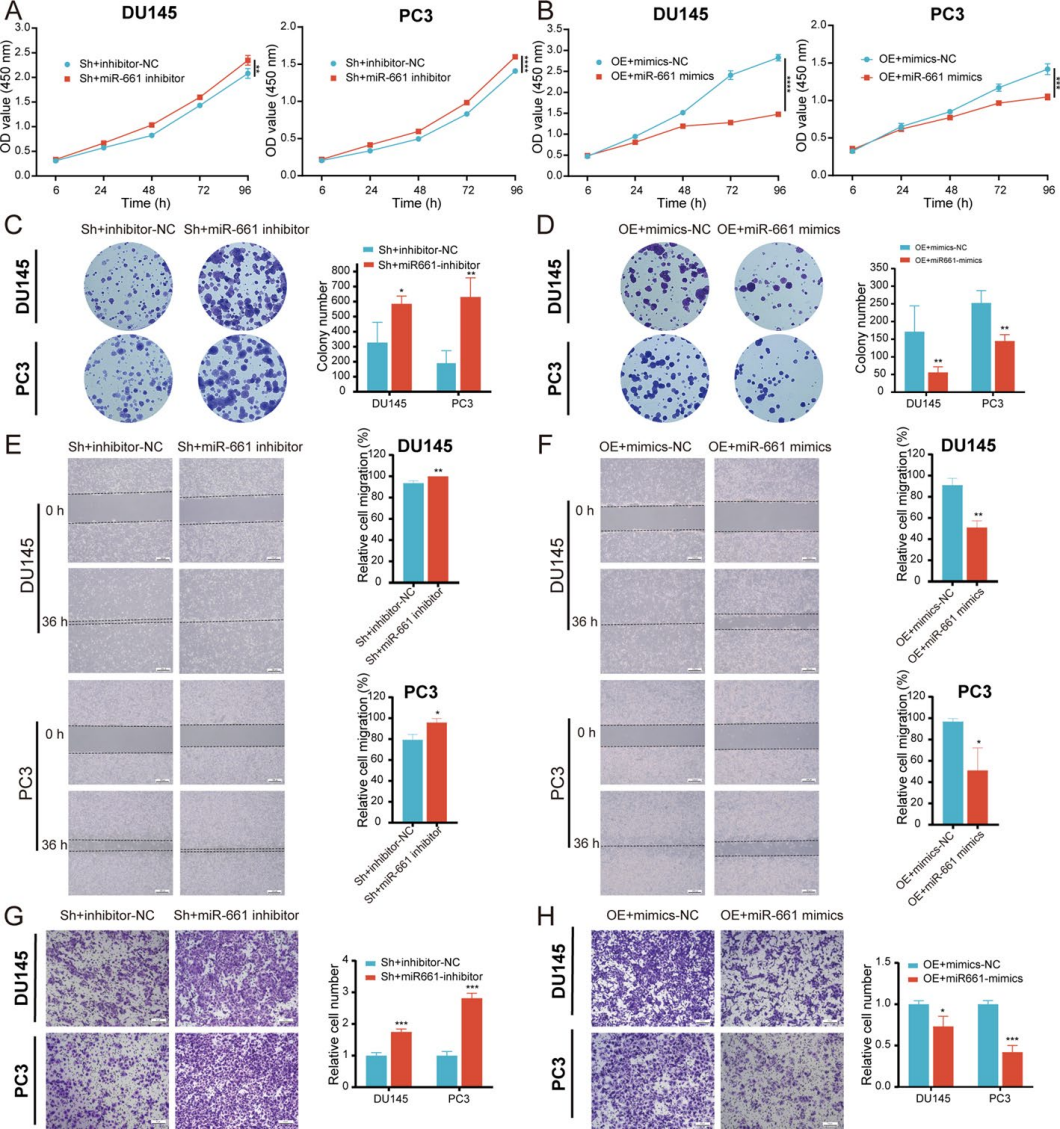
miR-661 reverses circGLIS3-mediated PCa progression. **A**–**D** In the CCK-8 and colony formation assays, miR-661 attenuated the inhibitory and facilitative effects of cell growth induced by silencing or upregulating circGLIS3, respectively. **E**–**H** The role of miR-661 on migration and invasion was evaluated in downregulating or overexpressing circGLIS3 DU145 and PC3 cells, respectively. The data were presented as mean ± SD. *p < 0.05, **p < 0.01, ***p < 0.001, and ****p < 0.0001

To investigate the effect of miR-661 on circGLIS3-mediated PCa progression, we downregulated or upregulated miR-661 using the inhibitor and mimics in circGLIS3-overexpressing and circGLIS3-silenced PCa cells, respectively. In the CCK-8 and plate colony formation assays, miR-661 downregulation attenuated the decrease in the growth of PCa cells with stable knockdown of circGLIS3 (Fig. 6A and C). Likewise, miR-661 upregulation impaired the promoting effect of cell proliferation in circGLIS3-overexpressing DU145 and PC3 cells (Fig. 6B and D). In line with the above, the miR-661 inhibitor weakened the reduction of PCa cell migration and invasion mediated by the silence of circGLIS3 (Fig. 6E and G). Furthermore, miR-661 mimics also reversed the promotion of cell migration and invasion caused by circGLIS3 overexpression (Figs. 6F and H). Therefore, those results all showed that miR-661 reverses circGLIS3-induced PCa progression.

### CircGLIS3 regulates MDM2/p53 pathway via sponging miR-661

**Fig. 7:**
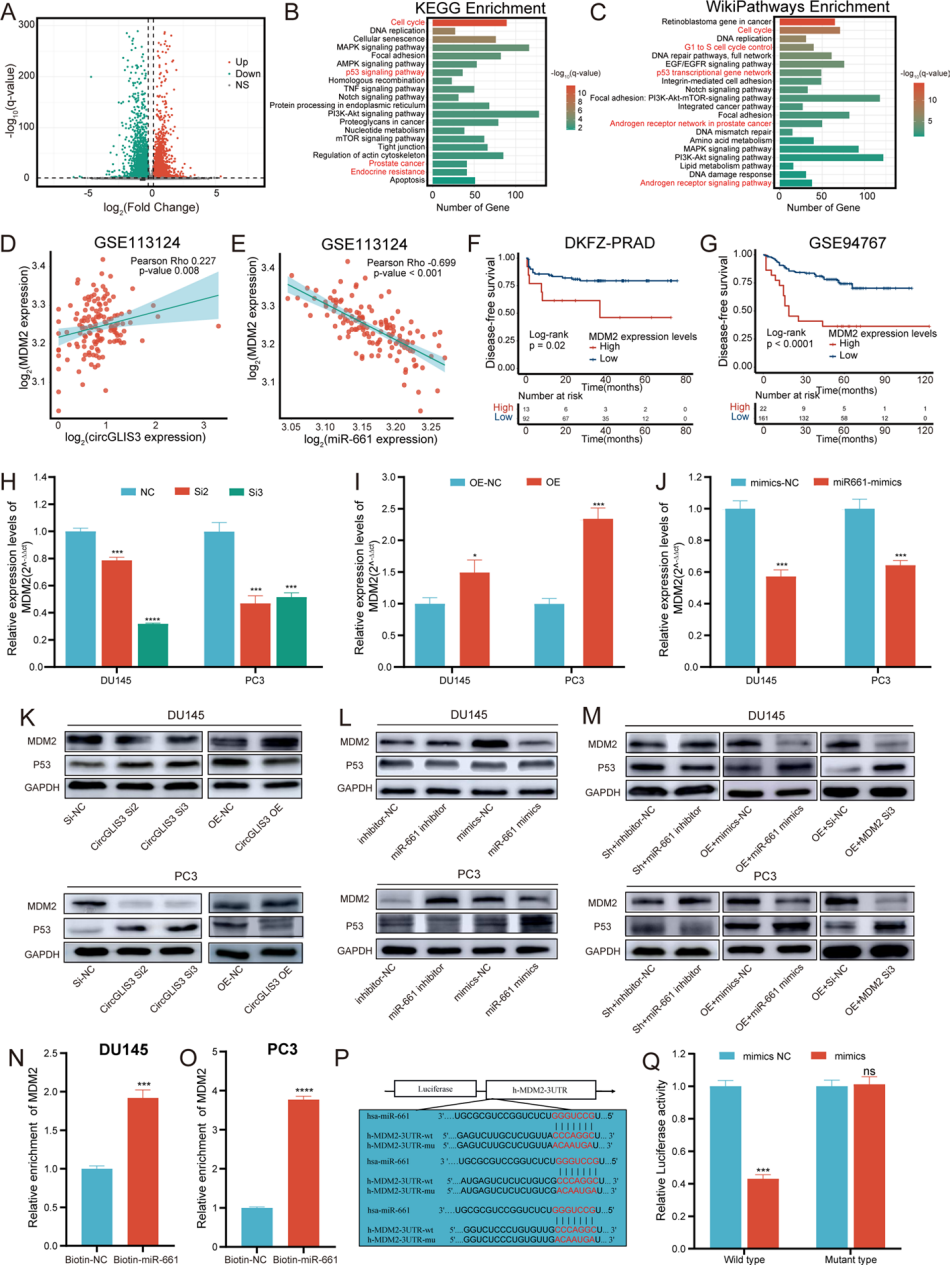
MDM2 acts as the target gene of miR-661. **A** Volcano plot of differentially expressed genes. **B**, **C** Kyoto Encyclopedia of Genes and Genomes (KEGG) and WikiPathways enrichment. **D**, **E** Pearson correlation between circGLIS3 or miR-661 and MDM2 in GSE113124. **F**, **G** The Kaplan–Meier survival curve analysis with a log-rank test was conducted to assess the prognostic value of MDM2 in the DKFZ-PRAD and GSE94767 cohorts, respectively. **H**, **I** The relative expression of MDM2 was altered after silencing or overexpressing circGLIS3, respectively. **J** MDM2 expression was declined in PCa cells after transfection with miR-661 mimics. **K**, **L** In western blot assay, the relative MDM2, and p53 protein levels were detected in DU145 and PC3 cells transfected with circGLIS3-siRNA, plasmid or lentiviral, miR-661-inhibitor, and miR-661 mimics. **M** the relative levels changes of MDM2 and p53 proteins after transfection with miR-661 inhibitors, miR-661 mimics, and MDM2-siRNA in PCa cells stably silenced or overexpressing circGLIS3. **N**, **O** miRNA pulldown assay was conducted to detect the enrichment level of circGLIS3. **P** MDM2 wild-type or mutated-type vectors were constructed following the illustration. **Q** The luciferase activities in HEK-293T cells were measured after cotransfection with MDM2 wild-type or mutated-type vectors and miR-661 mimics or scramble. The data were presented as mean ± SD. *p < 0.05, **p < 0.01, ***p < 0.001, and ****p < 0.0001

To identify the downstream genes of miR-661, RNA sequencing was performed to analyze differential expression genes between PC3 cells transfected with circGLIS3 siRNA and scramble. The results, as illustrated in Fig. 7A, were detailed in Supplementary Table S3. The enrichment analysis indicated that the signaling pathway mainly enriched in the cell cycle, p53 signaling pathway, AR signaling pathway, and endocrine resistance (Fig. 7B, C, Supplementary Fig. 2I, and Supplementary Table S3). Previous studies have highlighted that the p53 signaling pathway regulates the cell cycle and AR protein expression in PCa [21]. Specifically, MDM2, as a negative regulator, downregulates p53 by promoting ubiquitylation and proteasome-dependent degradation [22]. Notably, miR-661 has been reported to downregulate MDM2 and activate p53 pathway [23]. Silencing MDM2 reduced cell proliferation and induced apoptosis [24]. Based on these insights, we hypothesized that MDM2 acts as a target gene for miR-661 sponged by circGLIS3.

Pearson’s correlation analysis revealed a positive correlation between circGLIS3 and MDM2 in GSE113124, while miR-661 exhibited a negative correlation with MDM2 (Fig. 7D, E). Meanwhile, high MDM2 expression was notably associated with unfavorable disease-free survival in the DKFZ-PRAD and GSE94767 cohorts, respectively (Fig. 7F and G). Furthermore, silencing circGLIS3 notably downregulated MDM2, while circGLIS3 overexpression upregulated MDM2 (Fig. 7H and I). Similarly, miR-661 mimics reduced MDM2 expression in DU145 and PC3 cells (Fig. 7J). To validate this regulatory mechanism at the protein level, western blotting revealed consistent changes in MDM2 levels with circRNA silencing and overexpression, while p53 exhibited the reverse pattern (Fig. 7K). As shown in Fig. 7L, the miR-661 inhibitor increased MDM2 expression and reduced p53 levels, whereas its mimics induced opposite alterations. Besides, the miR-661 inhibitor attenuated MDM2 expression after the knockdown of circGLIS3. miR-661 mimics abolished MDM2 expression in DU145 and PC3 cells following overexpression of circGLIS3. MDM2 siRNAs reversed the elevation of MDM2 caused by circGLIS3 overexpression (Fig. 7M).

To validate whether miR-661 directly binds to the 3’UTR of MDM2, miRNA pull down assay demonstrated that MDM2 was significantly enriched to the biotin-labeled miR-661 probe in DU145 and PC3 cells (Fig. 7N and O). According to the potential binding sites, the mutated-type and wild-type MDM2 luciferase reporter plasmids were constructed as depicted in Fig. 7P. In the dual luciferase reporter assay, miRNA mimics significantly suppressed luciferase activity in cells transfected with the wild-type MDM2 plasmid, whereas it did not affect luciferase activity in cells with the mutant-type MDM2 plasmid (Fig. 7Q). In summary, circGLIS3 regulates MDM2 expression via sponging miR-661.

### MDM2 reverses circGLIS3-induced PCa progression

**Fig. 8:**
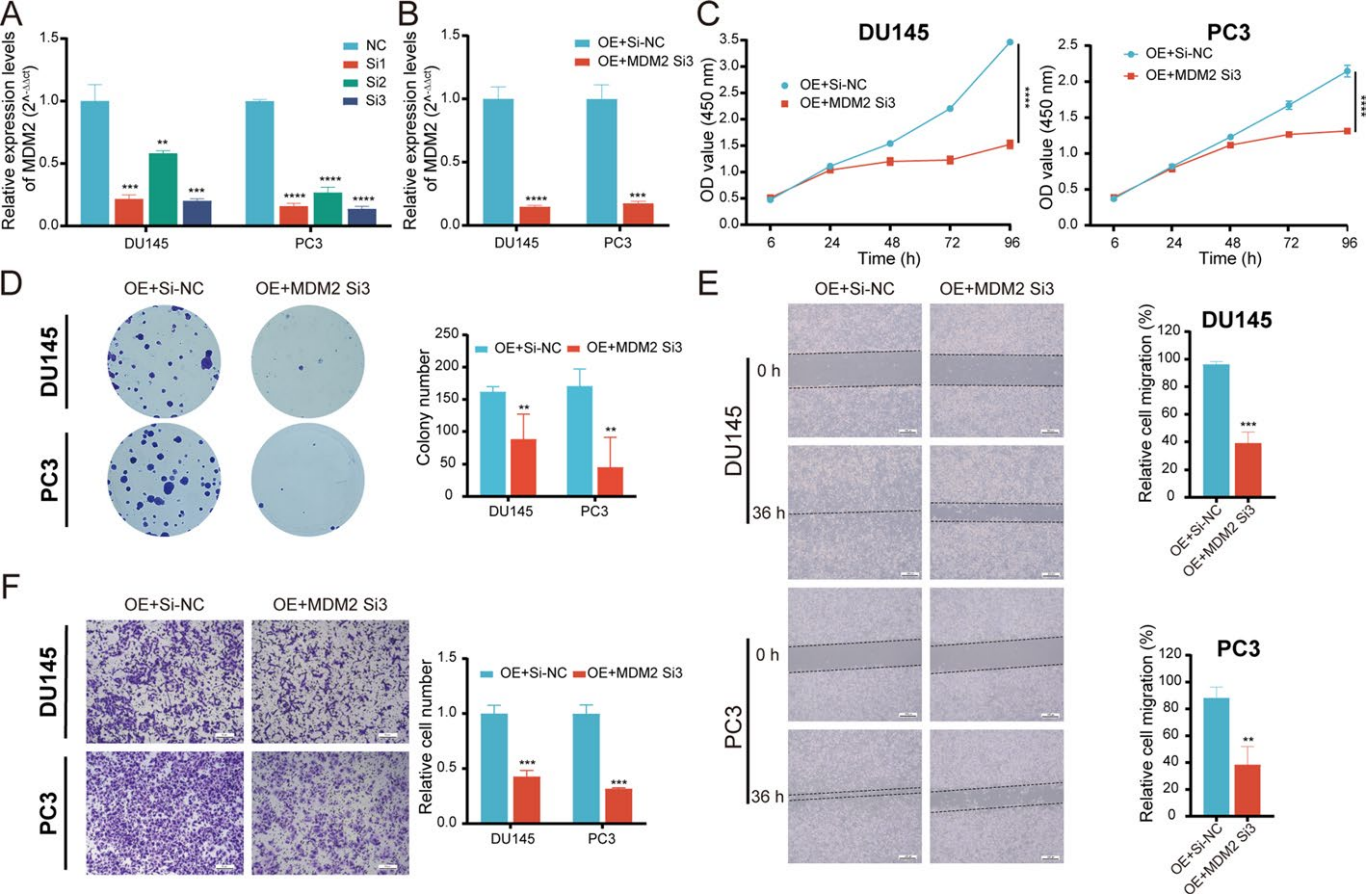
MDM2 reverses circGLIS3-induced PCa progression. **A** MDM2 relative expression levels after silencing it in DU145 and PC3 cells. **B** The qRT–PCR assay was used to assess the relative expression levels of MDM2 in PCa cells stably expressing circGLIS3 transfected with MDM2-siRNA. **C**, **D** The CCK-8 and colony formation assays showed that MDM2 weakened the promoting effect of cell proliferation in PCa cells overexpressing circGLIS3.**E**, **F** In wound healing and transwell assays, MDM2-siRNA inhibited the migration and invasive abilities of DU145 and PC3 cells upregulating circGLIS3. The data were presented as mean ± SD. *p < 0.05, **p < 0.01, ***p < 0.001, and ****p < 0.0001

To elucidate the effect of MDM2 on circGLIS3-induced PCa progression, rescue experiments were carried out. MDM2 siRNAs 3 dramatically reduced the expression of MDM2 (Fig. 8A). Meanwhile, it reversed the elevated MDM2 expression mediated by circGLIS3 overexpression in DU145 and PC3 cells (Fig. 8B). In CCK-8 assays, the silencing of MDM2 reverses the facilitation of cell proliferation induced by overexpressing circGLIS3 in PCa cells, which was also established by plate colony formation assays (Fig. 8C and D). Similarly, MDM2 knockdown effectively abrogated the enhanced migration and invasion capacity evoked by circGLIS3 overexpression in the transwell assays and wound healing assays (Fig. 8E and F). In general, circGLIS3 facilitates PCa progression through the MDM2/p53 signaling pathway.

### CircGLIS3 promotes PCa tumor growth in vivo

**Fig. 9:**
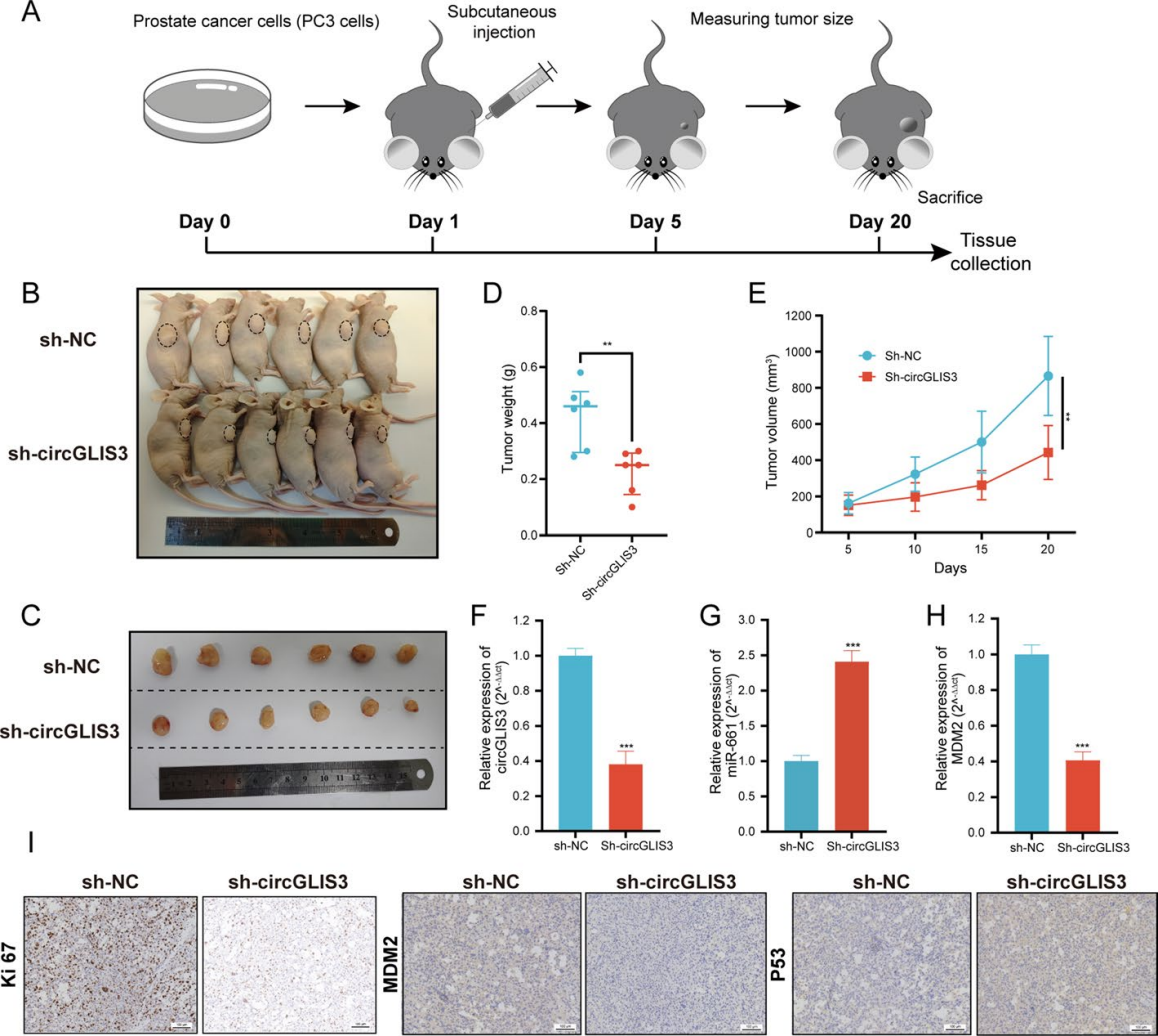
Knockdown of circGLIS3 inhibited tumor growth in vivo. **A** Schema of the subcutaneous tumor mouse model. **B**, **C** Images of nude mice and tumors from sh-NC and sh-circGLIS3 group, respectively. **D**, **E** Tumor weight and growth curve of tumor volume. **F**–**H** The relative expression of circGLIS3, miR-661, and MDM2 were detected by qRT–PCR, respectively. **I** The IHC assay detected the protein expression of Ki-67, MDM2, and p53 in both groups. The data were presented as mean ± SD. *p < 0.05, **p < 0.01, ***p < 0.001, and ****p < 0.0001

To further validate the biological function of circGLIS3 in *vivo*, the in vivo experiment was implemented with PC3 cells as shown in Fig. 9A. Notably, circGLIS3 silencing restrained the growth of transplanted tumors, which was in line with experimental results in vitro (Fig. 9B–E). Meanwhile, total RNA was isolated from transplanted tumors and subjected to qRT–PCR. The results indicated that circGLIS3 and MDM2 expression in the sh-circGLIS3 group were lower compared with the sh-NC group, whereas miR-661 was higher (Fig. 9F–H). In IHC assays, the expression levels of Ki-67 and MDM2 were significantly decreased after the knockdown of circGLIS3, while p53 was ascended (Fig. 9I). In conclusion, the in vivo results highlight the in vitro findings, further emphasizing the vital role of circGLIS3 in PCa progression.

### Knockdown of circGLIS3 improves PCa cell response for ARSI therapy such as enzalutamide in vitro and in vivo

**Fig. 10:**
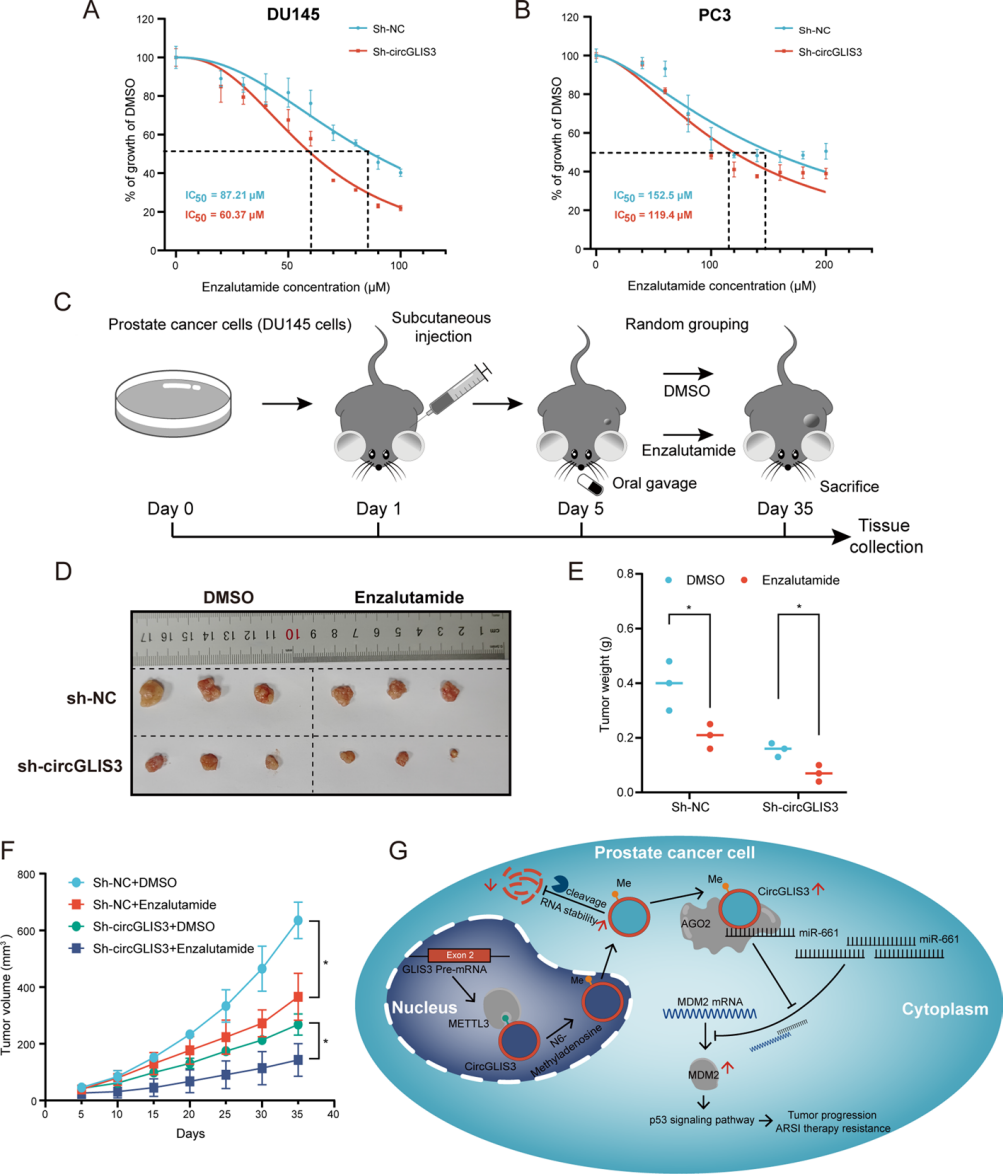
Silence of circGLIS3 enhances the response to enzalutamide in PCa. **A**, **B** The effects of enzalutamide on DU145 and PC3 cells after stable silencing circGLIS3. **C** Schema of in vivo experiment and oral administration of enzalutamide. **D**–**F** Tumor weight and tumor volume growth curve. **G** Illustrative schematic of circGLIS3 mechanism in PCa cell. The data were presented as mean ± SD. *p < 0.05, **p < 0.01, ***p < 0.001, and ****p < 0.0001

There were evidence of crosstalk between the AR and p53 signaling pathways in PCa [25]. Previous studies have revealed that MDM2 was involved in ARSI resistance in PCa [26, 27]. The enrichment analysis also suggested a potential involvement of circGLIS3 in endocrine resistance. Therefore, we speculated whether circGLIS3 might contribute to resistance to ARSI therapy in PCa. Enzalutamide, as novel generation ARSI, was utilize to evaluate PCa cell responses to ARSIs. In GSE118959 datasets, circCLIG3 was highly expressed in enzalutamide high-resistant LNCAP cells as shown in Supplementary Fig. 2J. The half-maximal inhibitory concentration (IC50) was measured to assess PCa cell responses to enzalutamide. With the drug concentration increases, the relative percentage of living DU145 and PC3 cells tends to decrease. PCa cells with stable circGLIS3 knockdown exhibited a more rapid decline than those with scramble, indicating that silencing of circGLIS3 enhances PCa cell responses to enzalutamide (Fig. 10A, B). 22RV1 cells as a CRPC cell line exhibit intrinsical resistance to enzalutamide. Thus, 22RV1 cells as an AR-positive enzalutamide-resistant cell line was employed to further elucidate the relationship between circGLIS3 and AR. After circGLIS3 knockdown, it showed similar results to the above (Supplementary Fig. 2K). In the western blot, its result indicated that knockdown of circGLIS3 decreased MDM2 and AR expression, while the expression of p53 was increased in 22RV1 cells (Supplementary Fig. 2L).

Moreover, to further ascertain if the sensitivity to enzalutamide was improved after downregulation of circGLIS3 in vivo, the mouse model of xenograft tumor was established according to schematic Fig. 10C. Tumor volume and weight were suppressed after silencing of circGLIS3, further restricted after oral administration of enzalutamide (Fig. 10D–F). In conclusion, circGLIS3 knockdown enhances PCa cell response to enzalutamide in vitro and in vivo by regulating the AR expression.

## Discussion

PCa, the most common malignancy in men, seriously threatens health [1]. Due to the heterogeneity of tumors, their natural course and treatment outcomes are highly variable. Thus, it is essential and imperative to explore novel mechanisms of tumorigenesis and therapeutic strategies. Furthermore, various drivers contribute to disease progression, including but not limited to, AR-dependent resistance, adapted resistance to the glucocorticoid receptor, and epigenetic alterations [28, 29]. CircRNA, as an emerging subclass of noncoding RNA, plays a key role in gene epigenetic regulation [16]. Accumulating evidence suggests that circRNA expression has been linked to various diseases, including cancer, neurodevelopmental processes, and autoimmune responses [12]. In light of this, we focus on the role of circRNA in promoting PCa progression.

Integrating bioinformatics analysis and large-scale qRT–PCR, the key candidate circRNA (circGLIS3) was selected. CircGLIS3 expression was significantly elevated in PCa tumor cells, as evidenced in PCa tissues. Our studies demonstrated that dysregulated circGLIS3 participates in tumorigenesis and progression. To elucidate its dysregulation, m^6^A, the most abundant type of methylation modification, was taken into consideration [12]. The m^6^A modification pattern of circRNA is distinct from that of mRNAs [18]. It regulates circRNA processing, splicing, translation, degradation, immunity, and tumors [12]. For instance, YTHDC1-induced m^6^A modification of circNSUN2 promotes the liver metastasis of colorectal cancer via modulating cytoplasmic export [30]. METTL3 mediated the m^6^A methylation stabilizes circCUX1, which further promoted tolerance to radiotherapy in hypopharyngeal squamous cell carcinoma [31]. In another study, m^6^A modification of circCCDC134 also enhanced its stability to facilitate tumor progression in cervical cancer [32]. Similarly, METTL3-mediated m^6^A methylation enhanced circGLIS3 stability, maintaining its overexpression in our studies. These reasons partly explain the upregulation of circGLIS3 in PCa.

A substantial body of evidence underscores the pivotal role of circRNAs in tumorigenesis and progression [33]. It exhibits tissue-restricted and developmental stage-specific expression patterns [33]. To date, various regulatory mechanisms, including sponging miRNAs, encoding proteins, binding with proteins, and modulating alternative splicing, have been proposed to elucidate the biological functions of circRNAs [34–38]. For example, circPTK2 binds to PABPC1 and enhances its ability to stabilize SETDB1 mRNA, thus promoting tumor metastasis and gemcitabine resistance in bladder cancer [39]. Circ_0086722 sponged miR-339-5p, regulating STAT5A to drive tumor progression in PCa [40]. CircGLIS3 has been reported as a tumor driver in several cancers, including bladder cancer, non-small cell lung cancer, and glioblastoma [41–43]. Our results revealed that circGLIS3 mainly localizes in the cytoplasm and directly bound with AGO2 in RIP assay, underscoring its role as a miRNA sponge. Interestingly, miRNA pulldown and luciferase reporter assays demonstrated that circGLIS3 directly bound with miR-661. miR-661 acts as a suppressor gene in melanoma, breast cancer, glioma, osteosarcoma, and gastric cancer, hindering tumor progression, which is in line with our findings [44–48]. Subsequently, the downstream gene MDM2 was identified and further validated by miRNA pulldown and luciferase reporter assays. MDM2, as a negative regulator of p53, plays a key role in PCa progression [49, 50]. MDM2 was overexpressed in various tumors, including PCa [24]. Its silence resulted in reduced cell proliferation, enhanced apoptosis, and inhibited migration and invasion in PCa, regardless of p53 status [24, 50]. Recently, therapeutic strategies targeting MDM2 have shown great promise in cancer, as evidenced by numerous clinical trials [51]. Our results also support its application in PCa management. Taken together, circGLIS3 may regulate the p53 signaling pathway partly via the miR-661/MDM2 axis, thereby promoting PCa progression.

In addition, our results revealed that the circGLIS3 silence synergistically enhances the antitumor effect of ARSI therapy both in vivo and in vitro. Previous studies have substantiated that antisense MDM2 downregulates AR expression and enhances the response of PCa cells to androgen deprivation [26, 27]. p53 could inhibit AR expression levels through combining with the p53 DNA binding site of the AR gene [52, 53]. Theoretically, silencing circGLIS3 activates the p53 signaling pathway, thereby suppressing AR expression. It results in increased sensitivity of PCa cells to enzalutamide. But these mechanisms between AR and MDM2 are under further investigation. The western blot assay suggested that the silencing of circGLIS3 causes a reduction in the MDM2 and AR level in 22RV1 cells. Our results endorse the concurrent application of antisense MDM2 and enzalutamide for PCa treatment. Meanwhile, the development of therapeutic strategies targeting circGLIS3 is promising. It is necessary to further investigate the other potential mechanism of circGLIS3 in induced enzalutamide-resistant cells using methods such as clustered regularly interspaced short palindromic repeats and CRISPR-associated protein 9 (CRISPR–Cas9) or patient-derived xenograft (PDX) mouse model in future studies. Overall, the downregulation of circGLIS3 improved the response for ARSI therapy, such as enzalutamide in PCa cells.

## Conclusions

Our study reveals a significant upregulation of circGLIS3 in PCa tissues and cells, closely correlated with BCR. METTL3-mediated m^6^A modification plays a vital role in stabilizing the expression of circGLIS3. Mechanically, circGLIS3 sponged miR-661 to regulate MDM2 expression, thus promoting PCa progression (Fig. 10G). Additionally, the knockdown of circGLIS3 enhances the sensitivity of PCa cells to enzalutamide. These findings provide novel insights into the mechanisms driving PCa progression, highlighting a potential targets for ARSI therapy.

### Supplementary Information

Supplementary Material 1. Figure 1 **A-D **The Cell Cycleand apoptosiswere assessed after silencing circGLIS3.

Supplementary Material 2. Figure 2 **A-B** The relative m^6^A levels of circGLIS3 after silencing METTL3 in DU145 and PC3 cells. **C-D** In GSE107299, miR-661 expression was lower in patients with higher PSA and more advanced T stage. **E-H** miR-661 expression was detected after PCa cells were transfected with miR-661 inhibitors and mimics. **I** Gene set enrichment analysis of p53 signaling pathway. **J** Relative expression levels of circGLIS3 in enzalutamide sensitive, low-resistant, and high-resistant LNCAP cells in GSE118959 dataset. **K** The antitumor effects of enzalutamide on 22RV1 cells after stable knockdowning circGLIS3. **L **Western blot assay revealed the relative alterations in MDM2, p53, and AR in circGLIS3 silenced 22RV1 cells. *p < 0.05, **p < 0.01, ***p < 0.001, ****p < 0.0001.

Supplementary Material 3. Table S1. Sequence information for all primers, oligo RNAs, and probes

Supplementary Material 4. Table S2. BCR-related circRNAs with FPKM > 0.5 and correlation analysis

Supplementary Material 5. Table S3. Results of gene differential expression and gene enrichment analysis

## Data Availability

The datasets used and/or analyzed during the current study are available from the corresponding author on reasonable request.

## Abbreviations

ADT: Androgen deprivation therapy
AR: Androgen receptor
ARSI: AR signaling inhibitors
BCR: Biochemical recurrence
CCK-8: Cell Counting Kit-8
cDNA: Complementary DNA
CircRNA: Circular RNAs
CPC-GENE: Canadian Prostate Cancer Genome Network
CRPC: Castration-resistant prostate cancer
DAB: Diaminobenzidine
FBS: Fetal bovine serum
FC: Fold change
gDNA: Genomic DNA
GEO: Gene Expression Omnibus
GTEx: And the Genotype-Tissue Expression
IC50: Half-maximal inhibitory concentration
IHC: Immunohistochemistry
m^6^A: *N*^6^-Methyladenosine
meRIP: Methylated RNA immunoprecipitation
MiRNA: MicroRNA
mRNA: Messenger RNA
NC: Negative control
PCa: Prostate cancer
PDX: Patient-derived xenografts
qRT–PCR: Quantitative real-time polymerase chain reaction
RIP: RNA immunoprecipitation
SD: Standard deviation
shRNA: Short hairpin RNAs
SiRNA: Small interfering RNA
TCGA: The Cancer Genome Atlas
