# Supplementary figures and images for: METTL3-mediated m6A modification of circGLIS3 promotes prostate cancer progression and represents a potential target for ARSI therapy
Source: Cell Mol Biol Lett. 2024 Aug 14;29:109. doi: 10.1186/s11658-024-00628-z (PMC11325714; doi:10.1186/s11658-024-00628-z)

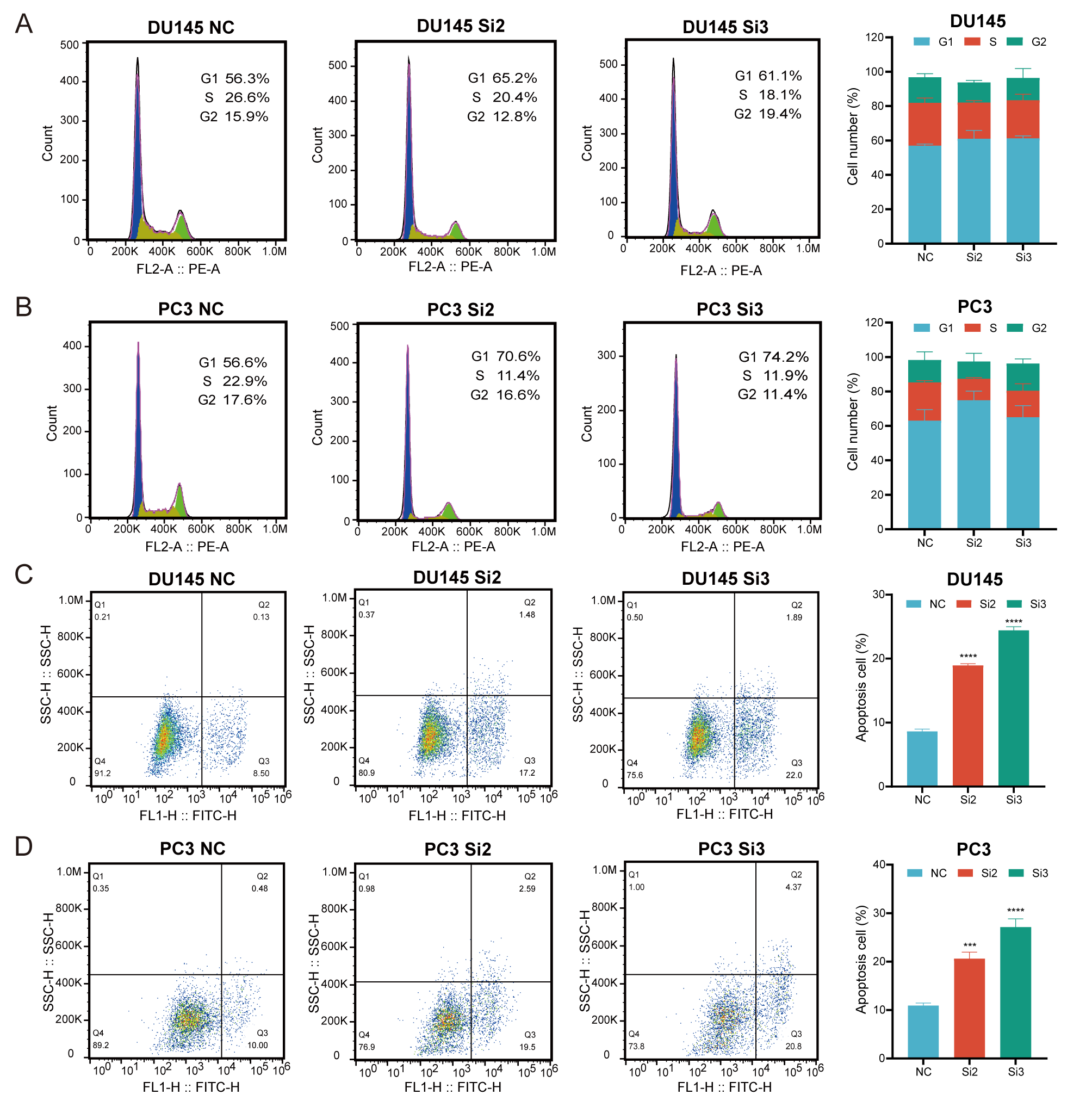

Supplement: Supplementary file 1 — Supplementary Material 1. Figure 1 A-D The Cell Cycleand apoptosiswere assessed after silencing circGLIS3. [file 11658_2024_628_MOESM1_ESM.tif]

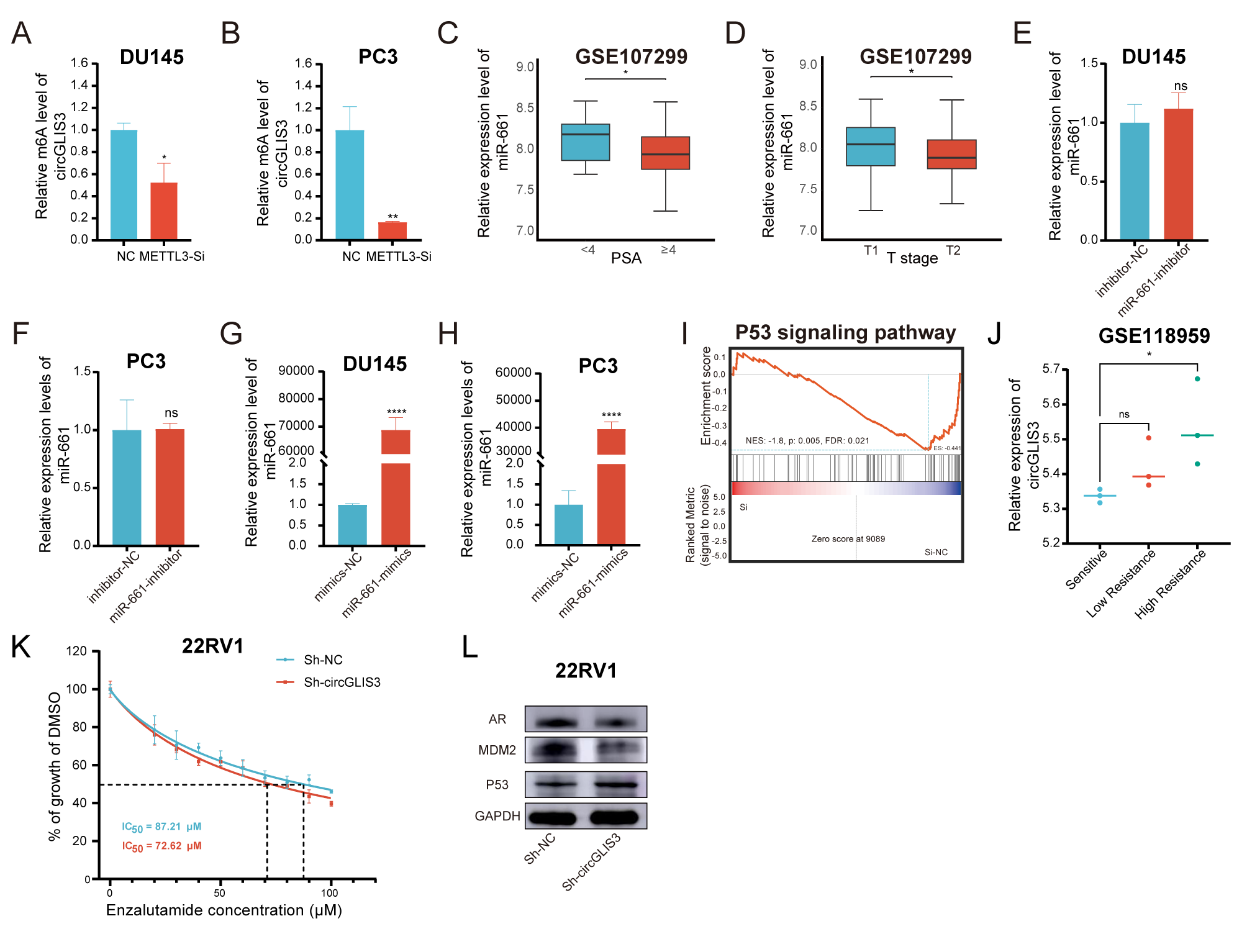

Supplement: Supplementary file 2 — Supplementary Material 2. Figure 2 A-B The relative m6A levels of circGLIS3 after silencing METTL3 in DU145 and PC3 cells. C-D In GSE107299, miR-661 expression was lower in patients with higher PSA and more advanced T stage. E-H miR-661 expression was detected after PCa cells were transfected with miR-661 inhibitors and mimics. I Gene set enrichment analysis of p53 signaling pathway. J Relative expression levels of circGLIS3 in enzalutamide sensitive, low-resistant, and high-resistant LNCAP cells in GSE118959 dataset. K The antitumor effects of enzalutamide on 22RV1 cells after stable knockdowning circGLIS3. L Western blot assay revealed the relative alterations in MDM2, p53, and AR in circGLIS3 silenced 22RV1 cells. *p < 0.05, **p < 0.01, ***p < 0.001, ****p < 0.0001. [file 11658_2024_628_MOESM2_ESM.tif]
